# A robust pipeline with high replication rate for detection of somatic variants in the adaptive immune system as a source of common genetic variation in autoimmune disease

**DOI:** 10.1093/hmg/ddy425

**Published:** 2018-12-12

**Authors:** Lies Van Horebeek, Kelly Hilven, Klara Mallants, Annemarie Van Nieuwenhuijze, Tiina Kelkka, Paula Savola, Satu Mustjoki, Susan M Schlenner, Adrian Liston, Bénédicte Dubois, An Goris

**Affiliations:** 1 KU Leuven, Department of Neurosciences, Laboratory for Neuroimmunology, Leuven, Belgium; 2 VIB & KU Leuven Center for Brain and Disease Research, VIB, KU Leuven, Leuven, Belgium; 3 KU Leuven, Department of Microbiology and Immunology, Leuven, Belgium; 4 Hematology Research Unit Helsinki, University of Helsinki, Department of Hematology, Helsinki University Hospital Comprehensive Cancer Centre, FIN‐00290 Helsinki, Finland; 5 Department of Clinical Chemistry and Hematology, University of Helsinki, Helsinki, Finland; 6 Department of Neurology, University Hospitals Leuven, Leuven, Belgium

## Abstract

The role of somatic variants in diseases beyond cancer is increasingly being recognized, with potential roles in autoinflammatory and autoimmune diseases. However, as mutation rates and allele fractions are lower, studies in these diseases are substantially less tolerant of false positives, and bio-informatics algorithms require high replication rates. We developed a pipeline combining two variant callers, MuTect2 and VarScan2, with technical filtering and prioritization. Our pipeline detects somatic variants with allele fractions as low as 0.5% and achieves a replication rate of >55%. Validation in an independent data set demonstrates excellent performance (sensitivity > 57%, specificity > 98%, replication rate > 80%). We applied this pipeline to the autoimmune disease multiple sclerosis (MS) as a proof-of-principle. We demonstrate that 60% of MS patients carry 2–10 exonic somatic variants in their peripheral blood T and B cells, with the vast majority (80%) occurring in T cells and variants persisting over time. Synonymous variants significantly co-occur with non-synonymous variants. Systematic characterization indicates somatic variants are enriched for being novel or very rare in public databases of germline variants and trend towards being more damaging and conserved, as reflected by higher phred-scaled combined annotation-dependent depletion (CADD) and genomic evolutionary rate profiling (GERP) scores. Our pipeline and proof-of-principle now warrant further investigation of common somatic genetic variation on top of inherited genetic variation in the context of autoimmune disease, where it may offer subtle survival advantages to immune cells and contribute to the capacity of these cells to participate in the autoimmune reaction.

## Introduction

Somatic variants are genetic alterations that are not inherited but arise in particular cell subsets over time (1). The presence of these somatic variants may only be apparent when they provide the altered cells with a survival or proliferative advantage, possibly in the context of disease (1). The pathological role of somatic variants has long been recognized in cancer. Recently, multiple research groups have shown that somatic variants linked to cancer are not rare events, as a substantial proportion of the general population carry somatic variants in blood cells that may cause clonal hematopoietic expansion, and the occurrence of these variants is age related (2–5). Individuals carrying such variants do not have obvious symptoms but present with a more than 10-fold increased risk of developing hematological cancers, an increased mortality rate but also an approximately 2-fold increased risk of coronary heart disease and stroke, which are presumed to have an inflammatory component (3,4). Indeed, the contribution of somatic variants to diseases beyond cancer, including autoimmune, autoinflammatory and neurological disorders, is increasingly being uncovered (6–10). For example, somatic variants in established disease-associated genes have been implicated in the development of chronic infantile neurological, cutaneous, articular syndrome (*NLRP3*) (6), autoimmune lymphoproliferative syndrome (*TNFRSF6*) (7) and Alzheimer’s disease (*PSEN1, PSEN2*) (8,9). Furthermore, patients with large granular lymphocytic leukemia caused by somatic *STAT3* mutations present with rheumatoid arthritis (RA) four times more often than mutation negative patients (10,11).

**Table 1:** Baseline patient characteristics

**Patient**	**Gender**	**Age at disease onset**	**Disease duration** **(years)**	**Disease course**	**MSSS**	**OCB**	**IgG index**	**Treatment at sampling**	**Co-morbidities**
MS-1	F	24	16	BOMS	1.04	pos	0.65	Interferon-β	—
MS-2	M	36	18	BOMS	1.03	pos	1.55	Interferon-β	—
MS-3	M	31	30	BOMS	1.79	NA	NA	Never treated	—
MS-4	F	24	44	BOMS	NA	NA	NA	Interferon-β	—
MS-5	M	22	15	BOMS	0.71	pos	0.92	Interferon-β	—
MS-6	F	20	8	BOMS	4.96	neg	0.83	Fingolimod	—
MS-7	M	40	9	BOMS	0.24	NA	0.56	Interferon-β	—
MS-8	F	45	22	PPMS	3.65	pos	1.09	Never treated	Breast + kidney cancer, HT
MS-9	F	29	14	BOMS	1.92	pos	1.92	Interferon-β	—
MS-10	M	42	6	BOMS	2.01	pos	1.31	Interferon-β	—

MSSS: multiple sclerosis severity score; OCB: oligoclonal band status; IgG: immunoglobulin G; F: female, M: male; BOMS: bout onset MS; PPMS: primary progressive MS; pos: positive; neg: negative; HT: Hashimoto thyroiditis; NA: not available.

Despite these established examples, translating strategies for the identification of somatic variants from the cancer field to autoimmune diseases remains challenging (12). Somatic variants in cancer are typically identified by the tumor-normal design in which tumor tissue is compared to non-cancerous tissue from the same individual. Translation to the autoimmune field requires careful selection of cell types that can act as ‘target’ (instead of tumor) and ‘reference’ (instead of normal) cell type. Once the relevant immune cell types in autoimmune diseases have been chosen, two additional challenges occur. First, somatic variants exert more subtle proliferating capacities than seen in cancer and are present in a relatively small subset of immune cells. Hence, substantially lower allele fractions, as low as 1%, are expected in immune diseases compared to tumors. Whereas tools available for cancer have good sensitivity to call somatic variants, positive predictive values remain unacceptably low for these low allele fractions (13). Second, the somatic mutation rate in non-cancer samples is much lower than in cancer, thereby causing artefacts, which are typically in the same low allele fraction range, to vastly outnumber somatic variants. Together, these two challenges mean that studies of somatic variants in autoimmune diseases are substantially less tolerant of false positives and that upon variant calling, bioinformatics algorithms need to incorporate refined filtering criteria in order to achieve high true positive or replication rates (12).

In the current study, we establish a pipeline for the detection of lowly abundant somatic variants that achieves high replication rates and validate it in an independent data set. As a proof-of-principle, we subsequently apply this pipeline to the autoimmune disease multiple sclerosis (MS, OMIM entry 126200).

**Figure 1:**
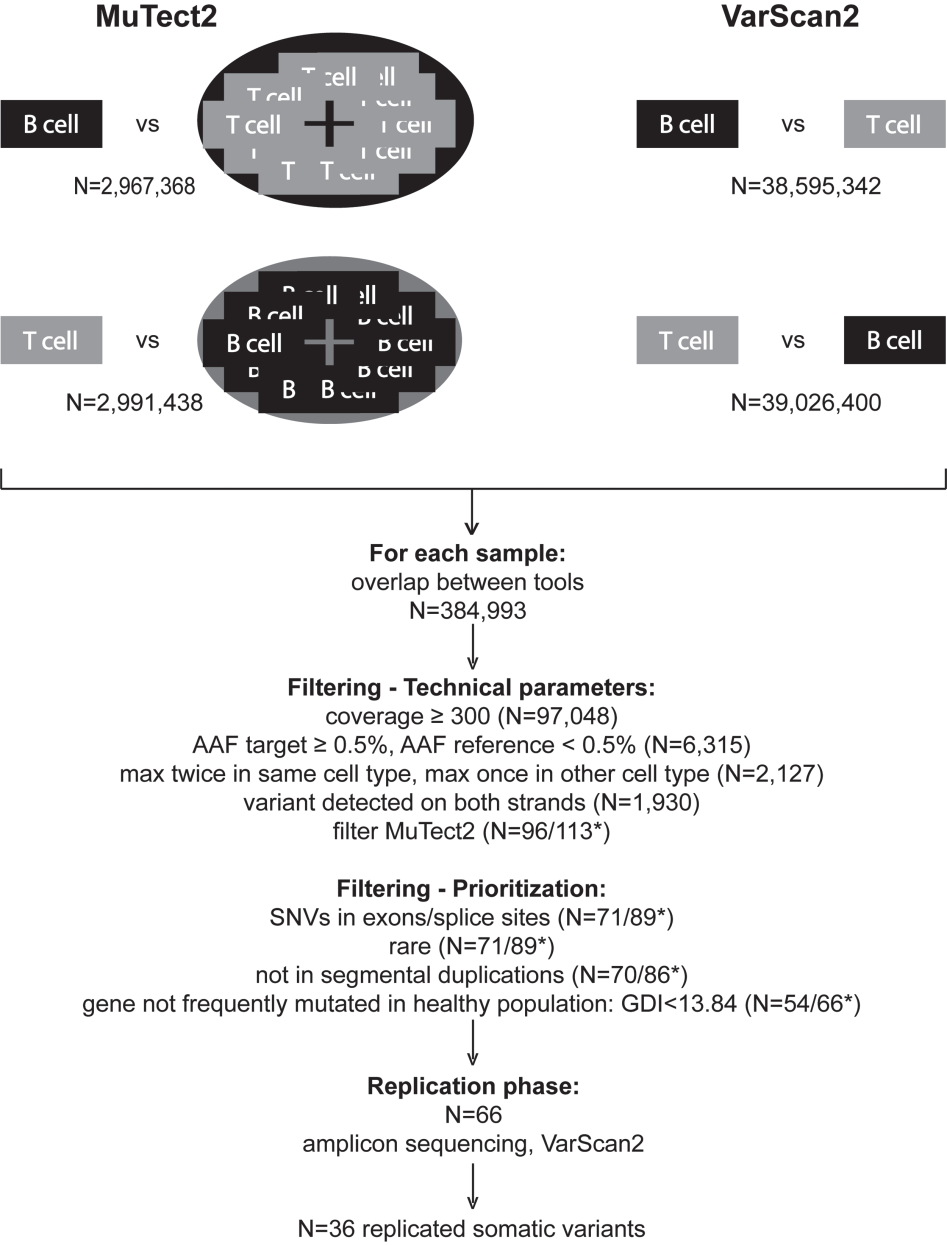
Pipeline for the detection of somatic variants in autoimmune diseases based on the overlap of variant callers MuTect2 and VarScan2. AAF: alternate allele fraction; GDI: gene damage index; *N*: total count; ^*^: default/adapted MuTect2 filters (original/final pipeline).

## Results

### Establishment of a pipeline for the detection of somatic variants in autoimmune disease

We hypothesized that somatic variants may act as a ‘second hit’ on top of a background of a high but in itself insufficient load of inherited risk variants. Hence, we calculated the genetic burden of 11 HLA and 110 non-HLA MS risk variants as described previously (14) and selected 10 MS patients with a genetic burden higher than the 95% percentile in controls (clinical details given in Table 1). As the adaptive immune system plays a key role in MS pathogenesis (15–17), we selected B and T cells for investigation. Using fluorescence-activated cell sorting (FACS), a median of 188 711 (range: 19 154–571 051) CD19^+^ B cells and 144 297 (range: 48 245–580 728) CD3^+^ T cells were isolated for each patient, with a purity of >95% for all cell types (Supplementary Materials, Fig. S1 and Table S1). We used targeted high-throughput sequencing to screen the DNA obtained from isolated B- and T-cell subsets from MS patients for somatic variants. We sequenced the coding regions of *N* = 5899 genes that we defined as candidates for somatic variants. These genes were either known Mendelian disease genes, in which variants likely have a large impact, or known MS risk genes (16). After quality control, we obtained a median sequencing depth of 342× (range: 163×–428×), with no obvious difference between CD19^+^ B cells (median: 334×; range: 163×–411×) and CD3^+^ T cells (median: 349×; range: 205×–428×).

B and T cells form two distinct lineages of the adaptive immune system. Somatic variants affecting specific lineages tend to be seen in non-lymphoproliferative disorders, whereas variants—even the same variants—present in a broader hematopoietic lineage give rise to Mendelian childhood immune disorders (6–10,18,19). Our approach to mutually compare B and T cells allowed the exclusion of germline variants and variants occurring in hematopoietic progenitor cells.

We performed somatic single-nucleotide variant (SNV) calling with a custom pipeline (Fig. 1) combining two established somatic variant callers, Mutect2 (20) and Varscan2 (21). Both variant callers have very good sensitivity to call somatic variants, but positive predictive values for each remain low in particular for variants with low allele fractions (13,22,23). It has been demonstrated that when calls of different variant callers agree, they tend to agree for true positive variants, and when they disagree, these variants are more likely to be false positives (24). Hence, we first focused on the overlap in called variants between both tools, which reduced the very large number of putative variants with 1–2 orders of magnitude (Fig. 1).

The two variant callers are furthermore complementary in providing additional technical filtering to distinguish true from false positive variants. Mutect2 has nine specific built-in filters, as well as the ‘panel of normal’ option, to compare the target cell type of each patient (B or T) to a ‘panel of normals’ consisting of the pooled variants found in the reference cell type (T or B) of all patients. Varscan2 provides an extensive and detailed output, which enables additional filtering on aspects such as sufficient coverage (≥300×), plausible alternate allele fraction (AAF) (≥0.5% in target and <0.5% in reference cell type) and no evidence of strand bias (alternate allele detected on both strands) (Fig. 1).

Similar to strategies typically applied for second generation sequencing of germline variants, we subsequently prioritized putative somatic variants for exonic or splice site SNVs that are rare (≤1%) in public databases of germline variants and not located in segmental duplications or in genes known to mutate frequently in the general population [gene damage index (GDI) < 13.84] (25) (Fig. 1).

### Validation of the pipeline in an independent data set demonstrates high replication rate

In order to obtain an appropriate validation of our pipeline, we applied it on the targeted sequencing data from an independent data set of 25 patients with RA (OMIM entry 180300). Previously published data for this RA data set (19) showed a replication rate of 14.3% for the used pipeline based on 1 variant caller (VarScan2) and provided 7 known true positive and 42 known false positive somatic SNVs for comparison in our study. For this comparison, we scaled up cut-off values in our pipeline to the larger sample size and the use of more specific T-cell subsets instead of T cells (Supplementary Material, Table S2). We first compared the identification of putative somatic variants (variant calling and technical filtering criteria), followed by the evaluation of the prioritization strategy.

**Table 2:** Overview of replicated somatic variants

**Chr**		**Position**	**Ref**	**Alt**	**Gene**	**AA change**	**Cell**	**AAF screen (%)**	**AAF repl (%)**	**P repl**	**CADD**	**MSC**	**GERP++**	**Kaviar**	**COSMIC**
**MS-1**															
^*^	2	24952447	C	T	*NCOA1*	P988P	T	2.00	0.57	9.56E-207	13.15	3.31	4.98	6.50E-6	−
	3	48508943	C	G	*TREX1*	L352 V	T	2.78	2.53	1.05E-149	23.50	5.61	1.35	0	−
^*^	11	8734271	G	A	*ST5*	R247C	T	0.75	1.12	1.42E-272	35.00	17.32	5.28	6.50E-6	−
	19	49703983	G	A	*TRPM4*	R611H	T	1.60	0.85	2.67E-87	30.00	0.09	4.57	5.82E-5	−
**MS-3**															
^*^	1	93202076	T	C	*EVI5*	T54A	T	0.98	1.14	8.66E-108	0.08	3.31	−0.13	1.29E-5	−
^*^	2	33590430	A	T	*LTBP1*	D1156V	T	0.70	0.80	3.71E-56	27.20	3.31	5.67	0	−
^*^	3	46244852	C	G	*CCR1*	R318T	B	1.22	1.41	5.80E-143	0.24	3.31	−2.00	0	−
^*^	11	63403722	T	C	*ATL3*	N294S	T	2.51	2.72	0	23.10	27.30	5.55	4.53E-5	−
^*^	11	128680557	A	G	*FLI1*	K152E	T	1.16	1.45	2.24E-291	23.90	23.30	3.89	0	−
^*^	12	123812503	G	C	*SBNO1*	G456G	T	0.79	5.58	0	3.43	3.31	2.02	0	−
^*^	16	67116210	A	T	*CBFB*	E165V	B	1.85	2.79	2.10E-163	33.00	15.45	5.54	0	−
^*^	Y	16734258	C	T	*NLGN4Y*	R87W	B	1.07	0.77	1.33E-06	27.10	15.26	0.54	0	−
**MS-4**															
^*^	6	74073541	G	A	*KHDC3L*	Q204Q	T	5.63	3.77	0	0.97	22.30	1.63	0	−
^*^	16	88504410	T	C	*ZNF469*	L3483P	T	1.25	4.28	2.38E-06	3.60	0.00	0.28	0	−
**MS-8**															
	2	135888230	G	A	*RAB3GAP1*	R392Q	T	0.70	0.51	4.90E-10	22.70	8.10	3.43	3.23E-5	+
	6	31631776	G	C	*GPANK1*	S160R	T	18.33	13.88	0	8.47	3.31	3.38	0	−
	6	149700524	T	C	*TAB2*	L491 L	T	2.24	2.55	0	0.04	0.01	−6.98	0	−
	7	47409021	G	A	*TNS3*	R408C	T	0.98	0.64	1.99E-197	23.50	3.31	4.91	1.29E-5	−
	8	135521904	C	A	*ZFAT*	S1088S	B	0.63	0.69	1.19E-88	20.70	3.31	−11.6	0	−
	16	67517193	C	T	*AGRP*	A37T	B	2.15	1.88	2.52E-21	22.20	3.31	4.20	0	−
	21	15538710	G	C	*LIPI*	P236A	T	15.54	16.58	0	23.80	3.31	5.48	6.50E-6	−
**MS-9**															
	1	240071069	C	T	*CHRM3*	F106F	T	7.47	3.67	0	9.42	3.31	4.69	0	−
	2	97427889	A	T	*CNNM4*	M385 L	B	1.20	1.68	1.16E-245	18.11	0.00	5.19	0	−
	5	133481460	T	C	*TCF7*	D253D	T	1.12	0.81	0	6.13	5.37	1.39	3.88E-4	−
	16	61891025	G	A	*CDH8*	T222I	T	3.77	3.36	4.32E-252	29.30	3.31	5.88	0	−
	X	29972647	G	T	*IL1RAPL1*	D404Y	T	1.30	0.79	4.57E-276	30.00	32.00	5.72	0	+
**MS-10**															
	2	141533745	C	T	*LRP1B*	G1808R	T	2.72	1.97	0	34.00	5.45	5.69	6.50E-6	−
	6	26508797	C	A	*BTN1A1*	R326R	T	1.51	1.58	0	10.95	3.31	2.10	3.23E-5	−
	7	103048325	C	T	*SLC26A5*	P287P	T	1.19	1.51	0	14.92	3.31	−3.99	1.94E-5	−
	7	122635510	T	G	*TAS2R16*	Q60P	T	3.76	3.89	0	23.20	3.31	3.42	0	−
	9	73477936	C	T	*TRPM3*	R117Q	T	0.52	0.64	8.75E-119	22.80	3.31	5.95	6.50E-6	+
	11	118373702	A	C	*KMT2A*	K2365 N	T	1.60	1.72	9.15E-33	18.97	26.10	4.03	0	−
	12	2622058	A	T	*CACNA1C*	D433V	T	2.98	3.83	0	28.40	0.07	4.43	0	−
	17	65026679	C	T	*CACNG4*	Y181Y	T	8.23	8.23	0	10.62	13.3	−4.15	3.88E-5	−
	19	37618680	A	C	*ZNF420*	N263H	T	0.62	0.61	3.24E-36	14.07	3.31	2.91	0	−
	X	17746076	G	T	*NHS*	D1086Y	B	0.92	0.48	1.52E-05	24.60	0.00	5.49	0	−

List of replicated somatic variants grouped per patient. ^*^: a second sample, obtained between 8 months and 2 years after the original sample, was used for the replication phase; Chr: Chromosome, Pos: position on hg19; Ref: reference allele, Alt: alternate allele, AA: amino acid change in the corresponding Gene (RefSeq ID of corresponding transcript in Supplementary Material, Table S5); AAF: alternate allele fraction in screening (screen) and replication (repl) phase; p repl: VarScan2 *P*-value in support of a somatic variant in the replication phase; CADD: phred-scaled combined annotation depletion score (predicted deleteriousness), MSC: mutation significance cut-off (gene-level lower limit of the 99% confidence interval of CADD scores); GERP: genomic evolutionary rate profiling score (score > 3 = conserved); Kaviar: frequency of the alternate allele in the Kaviar public database of germline variants; COSMIC: presence of the variant in the COSMIC70 database of somatic variants.

Our pipeline was able to correctly filter out 41/42 known false positive somatic variants (specificity of 98%). Whereas four known positive variants were correctly identified before prioritization (sensitivity of 57%), three others failed the technical filtering criteria, in particular, two Mutect2 filters related to the presence of adjacent or nearby events (*clustered events* or *homologous mapping event*). Overruling these filters in the RA data set correctly identified these known positive variants without wrongly calling any false positives. This suggests that whereas our pipeline already outperformed in terms of filtering out artefacts (high specificity and replication rate), it could further be improved in terms of sensitivity by future versions of MuTect2 under development or—as we further implemented here—by custom overruling of the two Mutect2 filters related to nearby events.

Our pipeline resulted in a replication rate of 87.5%, substantially higher than the 14.3% obtained in the original data set with one variant caller (19). A systematic analysis (Supplementary Material, Table S3) demonstrated that our combination of two variant callers and technical filtering criteria together substantially reduced the proportion of artefacts to true positive somatic variants in the context of autoimmune diseases. The vast majority (37/41) of these artefacts was removed with the help of the second variant caller MuTect2, i.e. not detected by MuTect2 or not passing the default MuTect2 filters. The remaining four variants failed our technical filtering criteria. A substantial subset of known false positives (75.6%) failed for more than one technical aspect (Supplementary Material, Table S4).

The identification of somatic variants was followed in our pipeline by a prioritization strategy, which deprioritized 2/7 true positive variants because of their location in genes with a high GDI. As expected on the basis of the concept of GDI and the experience with germline variants (25), these represent 28% of variants, whereas high GDI genes only correspond to 3.25% of genes sequenced, with similar observations in our MS data set (Supplementary Material, Table S5). Although these variants may technically be true variants, they receive lower prioritization for their biological importance in a disease context. This underscores the relevance of adding biological prioritization criteria to the technical filtering steps in the context of somatic variants, in parallel with current practices for rare germline variants.

### Proof-of-principle in MS: somatic variants occur predominantly in T cells in 60% of patients and persist over time

Application of our final pipeline (Fig. 1) to our MS study population resulted in a set of 66 putative and prioritized somatic variants (Supplementary Material, Table S6). For replication, CD3^+^ T cells and CD19^+^ B cells were isolated by FACS from a second blood sample obtained at the same time point as the sample for screening (for 41 variants) or a blood sample obtained on average 1 year later (for 25 variants). For each putative somatic variant, we performed amplicon sequencing with a median sequencing depth of 129 498 (range: 193–455 353). This high sequencing depth enabled us to use the VarScan2 *P*-value after Bonferroni correction (*P* < 5.88 × 10^−4^) together with the direction of effect (AAF target > reference) as confirmation determinants. We confirmed 36 of the 66 putative somatic variants, corresponding to a replication rate of 55% (Table 2). There was no difference in replication rate between amplicon sequencing in samples from the same or a later time point (*P* = 0.28). We compared sequencing and technical parameters between replicated and non-replicated variants (Supplementary Material, Fig. S2). The only parameter reaching significant association after correction for multiple testing was the target AAF in the screening phase.

**Figure 2:**
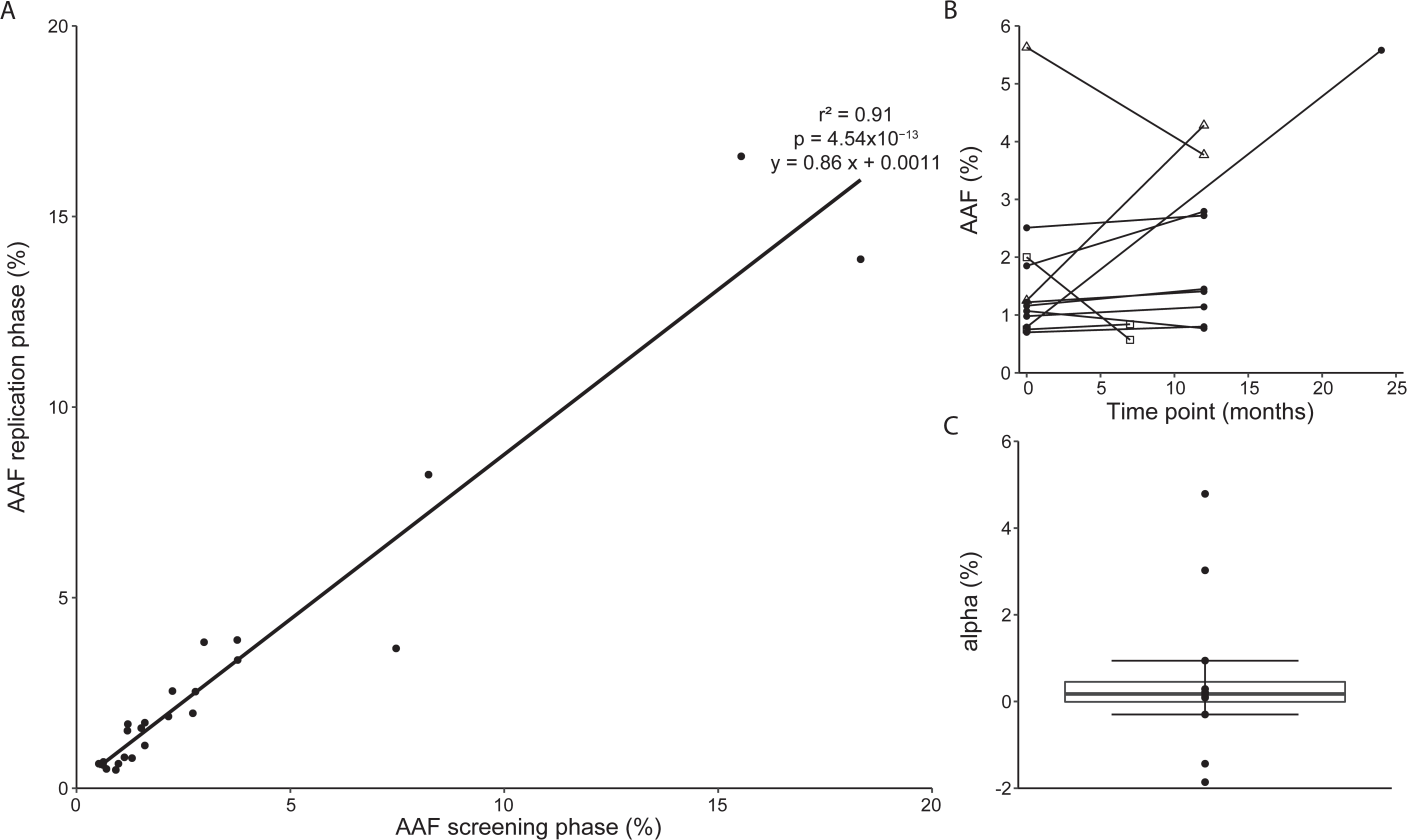
Somatic AAF: replication and evolution over time. For replicated somatic variants, the AAF is shown in the screening phase and in the replication phase using a second blood sample obtained at the same time point or a blood sample obtained on average 1 year later. (**A**) AAF correlates between screening and replication phases for samples from the same time point (*N* = 24 variants, *r*^2^ = 0.91, *P* = 4.54 × 10^−13^). (**B**) Somatic variants persist over time: evolution of AAF over time for longitudinal samples (*N* = 12 variants, time point 0 = screening phase, time point for replication phase on X-axis); patients indicated by symbols (square: MS-1, circle: MS-3, triangle: MS-4). (**C**) Clonal expansion rate (α) as change in AAF over time.

Despite the difference in sequencing depth, there was a strong correlation between the AAF determined from the screening and replication sequencing for samples from the same time point (*r*^2^ = 0.91, *P* = 4.54 × 10^−13^, 24 variants; Fig. 2A). For replication samples obtained on average 1 year later, allele fractions tended to increase, and a clonal expansion rate of 1.75% (range: 1.86–4.79%, *P* = 0.34) was estimated (Fig. 2B and C).

**Figure 3:**
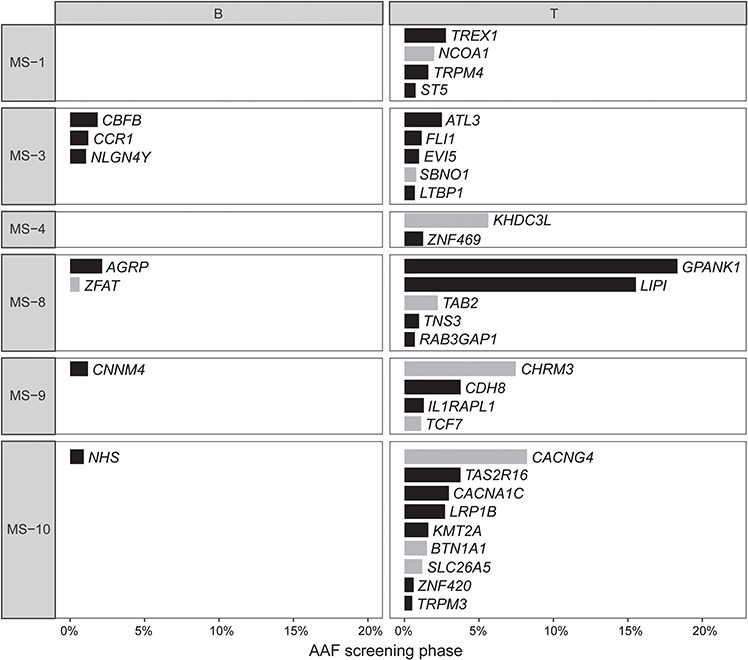
Clustering of somatic variants by cell type and by non-synonymous/synonymous effect. Somatic variants are observed in T cells of 60% of MS patients, with 40% of patients additionally carrying somatic variants in B cells. Synonymous variants (grey) co-occur with non-synonymous variants (black) (*P* = 0.0031). AAF: alternate allele fraction; gene names in italics; patients in which no somatic variant was identified (*N* = 4) not shown.

Confirmed somatic variants occurred predominantly in T cells, with 29 (80%) variants seen in T cells and 7 (20%) in B cells. We observed the presence of somatic variants in 60% (6/10) of MS patients. All 6 patients presented with multiple variants (2–10 variants per patient; Fig. 3), of which 2–9 variants occurred in T cells. Four of these patients additionally carried 1–3 variants in B cells (*P* = 0.07 for independence of variants in B and T cells).

Synonymous variants co-occurred with non-synonymous variants (*P* = 0.0031) and were only observed in patients carrying at least one non-synonymous variant. We observed no genes as mutational hotspots across this data set of 10 patients. There was no correlation between the variant count and patient’s age (*P* = 0.25) or disease duration (*P* = 0.87) at time of sampling.

### Somatic variants are enriched for being novel or rare and tend to be more conserved and damaging

**Figure 4:**
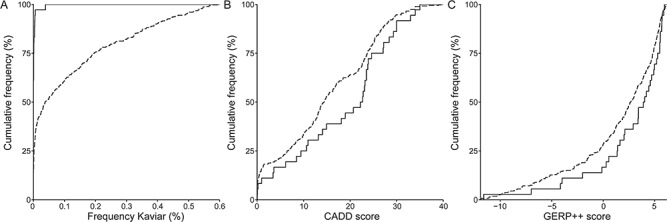
Somatic variant (full line) characteristics compared to matched germline variants (dashed line). (**A**) Somatic variants are enriched for being rare in public databases (Kaviar) (*P* = 9.80 × 10^−13^). (**B**) Somatic variants show a trend for being more damaging (CADD) (*P* = 0.057), and (**C**) the positions of somatic variants show a trend for being more conserved (GERP++) (*P* = 0.062). Non-parametric statistical tests (Kruskal–Wallis) were performed.

For comparison of variant characteristics, we compiled a set of matched germline variants observed in the same patients and filtered by the same criteria (Table 3). C>T transitions were most frequent, both among somatic and germline variants (*P* = 0.11), with a slightly larger proportion of the C>T transitions occurring at CpG positions (*P* = 0.58) (Supplementary Material, Fig. S3). A subset of 61.1% of somatic variants have never been observed in the Kaviar database of 77 781 exomes, substantially more than the 14.3% among the germline variants (*P* = 1.96 × 10^−9^). Similarly, somatic variants had significantly lower allele frequencies in public databases than germline variants (*P* = 9.80 × 10^−13^; Fig. 4A). The overlap with the catalogue of somatic variants in cancer database (COSMIC70) (26), on the other hand, was similar for somatic and germline variants, and none of the three overlapping somatic variants were related to immunoproliferative disorders. Somatic variants showed a trend for being more damaging as reflected by the phred-scaled combined annotation-dependent depletion (CADD) score (*P* = 0.057; Fig. 4B) and for being located at more conserved positions as indicated by the genomic evolutionary rate profiling (GERP) score (*P* = 0.062; Fig. 4C).

**Table 3:** Somatic variant characteristics compared to matched germline variants

		**Somatic (*N* = 36)**	**Germline (*N* = 378)**	***P***
Frequency	Novel	22 (61.11%)	54 (14.29%)	1.96 × 10^−9^
	Median (range)	0 (0–3.88 × 10^−4^)	0.0003752 (0–6.00 × 10^−4^)	9.80 × 10^−13^
COSMIC	Present	3 (8.33%)	17 (4.50%)	0.40
Pathogenicity	CADD > MSC:	29 (80.56%)	272 (72.00%)	0.33
	Median CADD (range)	22.45 (0.037–35.00)	14.40 (0.001–40.00)	0.057
Conservation	GERP > 3	22 (61.11%)	179 (47.35%)	0.12
	Median (range)	3.96 (−11.60–3.96)	2.66 (−11.90–6.17)	0.062

Frequency based on Kaviar database. *N*: total count; CADD: phred-scaled combined annotation-dependent depletion score; MSC: variant significance cut-off score; GERP++: genomic evolutionary rate profiling score.

**Table 4:** Accurate and sensitive ddPCR quantification of somatic variants: somatic variants are specific to T cell subsets

	***EVI5* p.T54A**			***LTBP1* p.D1156V**		
**Sample type**	**ALT**	**REF**	**ALT AF (%)**	**ALT**	**REF**	**ALT AF (%)**
CD3^+^ T cells	12.6	1000	1.26	—	—	—
CD8^+^ TEMRA cells	11.3	331	3.41	0	233	0
Remaining CD8^+^ T cells	0	247	0	0	184	0
CD4^+^ TEM cells	0	172	0	8.5	129	6.59
Remaining CD4^+^ T cells	0	197	0	0	89	0
CD19^+^ B cells	0.34	688.5	0.049^a^	—	—	—
Other immune cells	0.22	546	0.040^a^	—	—	—
Negative control	0.16	974.5	0.016^a^	—	—	—

Results for ddPCR measurements in DNA obtained from all T cells and isolated T-cell subsets, B cells and other (non-B and non-T) immune cells of MS-3*.* ALT and REF indicate the copies per μl of the alternate and reference allele, respectively, in a 20 μl ddPCR reaction. ALT AF (%) indicates the fraction of the alternate allele. TEMRA: T effector memory cells re-expressing CD45RA; TEM: T effector memory cells. ^a^Values below detection threshold (0.1%).

### Somatic variants cluster in same cell types

Intriguingly, the majority (80%) of the somatic variants was observed in T cells. We selected patient MS-3, the only patient with a somatic variant in an MS risk gene (*EVI5*), for a more detailed investigation of the cell subsets in which the somatic variants occurred. Hematological phenotyping of T cells of individual MS-3 showed a normal polyclonal T-cell receptor pattern (Supplementary Material, Fig. S4), indicating that the variants are not linked to the presence of a T-cell cancer, as this is typically accompanied by a monoclonal pattern. Extensive immunophenotyping data were available from a previous study (27) (Supplementary Material, Fig. S5). CD4^+^ cells (17.6% of T cells) were predominantly T effector memory (TEM) cells (47.61% of CD4^+^), whereas CD8^+^ cells (63.05% of T cells) were mainly effector memory T cells re-expressing CD45RA (TEMRA) (63.05% of CD8^+^). We hence sorted T cells from a second sample of PBMCs collected 1 year after the original sample into CD4^+^ TEM cells and all remaining CD4^+^ T cells and CD8^+^ TEMRA cells and all remaining CD8^+^ T cells. CD8^+^ TEMRA, CD4^+^ TEM and remaining CD4^+^ T cells each reached a purity of >96.5%, whereas for the remaining CD8^+^ T cells, a somewhat lower purity of 87.1% was obtained.

We established a droplet digital polymerase chain reaction (ddPCR) assay for the quantification of specific non-synonymous somatic variants. We set up a standard curve, determined the fraction of the alternate allele with ddPCR and observed a very high accuracy (*r*^2^ = 0.98) between duplicate measurements and a very strong correlation (*r*^2^ = 0.9997) between the expected (based on the input) and observed allele fractions (based on ddPCR output) (Supplementary Material, Fig. S6). This methodology was able to detect allele fractions as low as 0.1%.

Quantifying the somatic *EVI5* variant in T-cell subsets of MS-3 using ddPCR revealed an allele fraction in T cells of 1.26%, in line with the sequencing-based estimate of 1.05%. Within T cells, the somatic variant in *EVI5* was restricted to CD8^+^ TEMRA cells with an allele fraction of 3.41%, corresponding to ~7% of CD8^+^ TEMRA cells carrying this variant (Table 4; Supplementary Material, Fig. S7). The variant was below the detection threshold in other CD8^+^ T cells, CD4^+^ T cells, B cells and non-B non-T immune cells. By using molecular cloning, we could confirm that two additional non-synonymous somatic variants in *ATL3* and *FLI1* were shared by the same CD8^+^ TEMRA subset. A fourth variant, in *LTBP1*, was instead confined to CD4^+^ TEM cells, with an allele fraction of 6.6% or 13.2% of cells in this subset affected (Table 4; Supplementary Material, Fig. S7). In summary, non-synonymous variants in T cells of one individual were specific for T-cell subsets with a memory phenotype and the majority of variants clustered in the same subset.

## Discussion

We developed a pipeline that is able to identify somatic variants with low allele fractions in the peripheral blood of autoimmune disease patients with high replication rates and validated this pipeline using an independent data set. As a proof-of-principle, we applied this pipeline to MS and demonstrated that the majority of patients carries somatic variants, predominantly in T cells.

In a cohort of 10 MS patients, we identified 36 somatic variants with a median allele fraction of 1.63%, of which the vast majority (80%) occurred in T cells. Indeed, 60% of MS patients carried 2–9 variants in T cells and 40% carried 1–3 variants in B cells in addition to the variants in their T cells. These variants persisted and suggested clonal expansion over time but were not associated with age or disease duration. Our findings are in line with those of Valori *et al*. (28) who found 63% of MS patients carrying 1–4 somatic variants, 88% of them restricted to CD8^+^ T cells. Savola *et al*. recently reported somatic variants in T cells for another autoimmune disease, RA (19). The subset of patients affected was lower than for MS (20%), whereas the allele fraction was on average higher, and a more pronounced T-cell clonal proliferation pattern was seen (19). Together, these studies demonstrate that somatic variants in immune cells of autoimmune disease patients form a novel class of common genetic variation. As limitation, our study does not allow establishing whether somatic variants are disease specific or enriched in disease. Hence, upon our proof-of-principle, future perspectives include a more detailed comparison of both the occurrence and the characteristics of somatic variants in larger study populations of patients with different autoimmune diseases and of healthy controls. At current sequencing costs, a balance needs to be found between sequencing depth and the number of genes and cell types covered, inherently running the risk of missing relevant variants (19). Future extension to whole exome or even whole genome sequencing will allow investigating the contribution of somatic variants to autoimmune diseases such as MS even more extensively.

We subsequently moved beyond the detection of somatic variants to their characterization. Somatic variants in our study were enriched for being extremely rare in public databases of germline variants, with the majority (61%) even being novel, and showed a trend towards being more damaging and located at more conserved positions. Synonymous variants, which are more likely to be passenger variants resulting from immune cell proliferation (29), significantly co-occurred with non-synonymous variants, which are more likely to be driver variants (29). Several genes, such as *TREX1, FLI1, EVI5, IL1RAPL1*, are known for their role in autoimmunity, with *EVI5* being an established MS risk gene (16), whereas others, such as *CBFB, OPCML* and *KMT2A*, are involved in lymphoproliferative disorders (OMIM). Such variants may offer subtle survival advantages to immune cells and contribute to the capacity of these cells to participate in the autoimmune reaction. However, larger follow-up studies are required in order to conclude whether—and which—somatic variants in autoimmune diseases contribute to the disease process as drivers of an altered, proliferating or autoreactive phenotype or are a result of immune cell proliferation as passenger variants.

By in-depth analysis of one patient, we showed that three variants were shared by the same T-cell subset, CD8^+^ TEMRA cells, whereas the fourth affected CD4^+^ TEM cells. This corresponds to the data of Savola *et al.* (19) in supporting an effector memory phenotype for mutated T cells in autoimmune disease. Effector memory cells are increased in the peripheral blood of MS patients compared with controls and are exchanged between the peripheral blood and the central nervous system (27,30–32). Terminally differentiated memory cells increase with age (27,33) and after chronic infection, e.g. with the cytomegalovirus (34,35), but are still able to proliferate when surrounded by the appropriate stimuli and have been implicated in autoimmunity (36–38). Early work from the ‘90s using an *HPRT* assay suggested that somatic variants are enriched in autoreactive T cells from MS patients compared to non-autoreactive T cells or cells from healthy individuals (39,40). Further investigation will need to establish whether cells carrying somatic variants correspond to an altered, proliferating or autoreactive phenotype and whether they migrate to the central nervous system.

The experimental strategy and analysis pipeline in our current study enabled us to replicate 55% of the putative somatic variants, a rate that is substantially higher than that in other pilot studies in autoimmune diseases that were either unable to confirm candidate somatic variants (41) or obtained replication rates of 6–18% (19,28). In order to establish the performance, we applied our pipeline to a real-life, independent data set of RA patients (19). This validation confirmed the high replication rate (87.5%), as well as high specificity (98%), and demonstrated that our approach of combining different somatic variant callers and adding filtering criteria narrows down the list of identified variants while increasing the likelihood of correctly identifying somatic variants in the context of autoimmune diseases. This addresses the important challenge set out for translating tools for somatic variant identification from the cancer field to the field of autoimmune diseases (12); a challenge arising because in these diseases, artefacts typically outnumber true somatic variants, and both are in the low allele fraction range (12).

Although we applied our pipeline to MS as a proof-of-principle, it is applicable to other cell types and autoimmune or auto-inflammatory diseases, for which the role of somatic variants is increasingly recognized. The identification of a novel class of common genetic variants in autoimmune disease offers translational perspectives. Pathways and cell types affected by somatic variants in a patient may indicate which of the available treatments (e.g. affecting B or T cells) is most likely beneficial. For some somatic variants, it may even be possible to target the affected pathway itself, as examples such as *BRAF* or *STAT3* inhibitors for other autoimmune and neurological diseases illustrate (18,19). If cells carrying somatic variants—whether drivers or passengers—flag proliferating, autoreactive or pathogenic cells, the AAF could be used for follow-up over time (28). For this purpose, we have shown ddPCR to be a highly accurate and sensitive method for quantification of specific somatic variants with allele fractions as low as 0.1%. This may in particular indicate whether treatment effectively depletes cells and when a treatment changes or a subsequent treatment administration is required.

## Materials and Methods

### Study participants and sample collection

MS patients diagnosed based on the McDonald criteria (42) were recruited from the University Hospitals Leuven (UZ Leuven). The study has been approved by the Ethics Committee of the University Hospitals Leuven (ML4733), and written informed consent was obtained from all participants. All participants were of Caucasian descent, and extensive demographic and clinical data were collected through a questionnaire and medical records.

### Cell separation and DNA extraction

Peripheral blood samples were collected in 10 ml blood tubes containing ethylenediaminetetraacetic acid (EDTA, BD Vacutainer, Franklin Lakes, New Jersey). Peripheral blood mononuclear cells (PBMCs) were isolated using lymphoprep (Axis-Shield, Dundee, UK), resuspended in 1 ml fetal bovine serum (FBS, Tico Europe, Amstelveen, The Netherlands) containing 10% dimethylsulfoxide (Sigma-Aldrich, Saint-Louis, Missouri) and stored at −80°C until use.

The following antibodies, diluted in FACS wash buffer [20 mm phosphate buffered saline (PBS), 2% FBS, 1 mm EDTA, 0.001% NaN3 (Acros Organics, Geel, Belgium)], were used: FITC-labeled anti-human CD3 (1:125, eBioscience, Waltham, Massachusetts) for T cells; APC-eFluor® 780-labeled anti-human CD19 (1:50, eBioscience) for B cells and a mix of APC eFluor® 780-labeled anti-human CD45Ra (1:200, eBioscience), PE-cyanine 7-labeled anti-human CD8 (1:80, eBioscience), PE-labeled anti-human CD4 (1:25, eBioscience) and Alexa Fluor® 488-labeled anti-human CD197 (CCR7) (1:50, BioLegend, San Diego, California) for T-cell subsets. Other immune cells were defined as CD3^−^CD19^−^ immune cells.

PBMCs were thawed on ice and washed with 0.1% bovine serum albumin (Sigma-Aldrich) in PBS (Gibco—Thermo Fisher Scientific, Waltham, Massachusetts) containing 2 mm EDTA (Ambion, Waltham, Massachusetts) and centrifuged at 400 *g* for 7 min at 4°C. The cell pellet was washed in FACS wash buffer, centrifuged at 400 *g* for 7 min at 4°C, resuspended in 50 μl blocking buffer [Hu FcR Binding Inhibitor (eBioscience) 1:100 in PBS] and incubated on ice for 20 min. After another centrifugation step of 5 min at 400 *g* at 4°C, the cell pellet was resuspended in 25 μl stain mix. It was incubated on ice for isolation of CD19^+^ B cells and CD3^+^ T cells or at 37°C for the T-cell subset isolation for 30 min. Samples were centrifuged at 400 *g* for 5 min at 4°C, washed twice with FACS wash buffer and resuspended in FACS wash buffer containing 4′,6-diamidino-2-phenylindole (Thermo Fisher Scientific). Flow cytometry was performed with a BD FacsAriaIII instrument (BD Biosciences, Franklin Lakes, New Jersey).

DNA from isolated cells was extracted using an in-house extraction protocol. To the cell pellet obtained after centrifugation for 5 min at 400 *g* at 4°C, 250 μl SE buffer (75 mm NaCl, 25 mm EDTA), 2.5 μl proteinase K (20 mg/ml, VWR, Radnor, Pennsylvania) and 25 μl sodium dodecyl sulfate (Amresco, Radnor, Pennsylvania) 10% buffer were added, after which the sample was vortexed and incubated overnight at 37°C. The following day, 100 μl 5 m NaCl was added, and after shaking, the mixture was centrifuged for 5 min at 4700 rpm at 4°C. The supernatant was isolated and centrifuged twice for 5 min at 4700 rpm at 4°C. Additionally, one volume of isopropanol (Acros Organics) was added to the isolated supernatant, and the tube was turned until DNA was formed. Subsequently, the samples were centrifuged for 8 min at full speed, after which the supernatant was eliminated, and the DNA was washed with 500 μl 70% ethanol (VWR). After centrifuging this for 8 min at full speed, the ethanol was removed, and the DNA was air-dried. The resulting DNA was dissolved in 10/1 Tris-EDTA buffer [10 mm Tris (pH 8.0), 1 mm EDTA].

### Screening phase: identification of somatic variants

A SeqCap EZ XL panel (Roche, Basel, Switzerland) was designed containing the coding sequence of *N* = 5899 genes, i.e. known genes for Mendelian diseases (43) and known MS risk genes (16). From DNA obtained from isolated B and T cells of 10 MS patients, sample libraries were prepared using the KAPA Library Preparation Kit (Roche), enriched by hybridizing to the SeqCap EZ Choice XL pool and sequenced with the Illumina HiSeq2500 technology (PE100) (Genomics Core Facility, KU Leuven, Leuven, Belgium).

Sequence reads were aligned to the human Reference Genome Build (NCBI37/hg19) by BWA software (version 0.7.8) (44). Local realignment around indels and base recalibration were done using GATK (version 3.4) (45). Removing duplicates was performed as part of the variant calling (see below). Variant calling was performed with Mutect2 (version 3.5) and VarScan2 (version 2.3.9) in B and T cells of each patient, which were each in turn considered as ‘target’ or ‘reference’ tissue (20,21). With MuTect2, putative somatic variants were identified by comparing variants obtained in B cells of one patient to variants obtained in all T-cell samples (T panel of normals) and *vice versa*. With VarScan2, all deviations from the reference genome were listed for each sample and compared between the B- and T-cell samples from the same individual. The commands used in this study are listed below.

#### Mutect2 (default settings)

Preparing vcf file per cell type per patient based on bam file, including removal of duplicates:

java -jar GenomeAnalysisTK.jar -T MuTect2 -R human_g1k_v37.fasta -I:tumor patient1_celltype1.bam --artifact_detection_mode -nct 20 -o variants_patient1_celltype1_PON.vcf.

Combining variants per cell type in panel of normals:

java -jar GenomeAnalysisTK.jar -T CombineVariants -R human_g1k_v37.fasta -V variants_patient1_celltype1_PON.vcf -V variants_patient2_celltype1_PON.vcf -V variants_patientN_celltype1_PON.vcf -minN 1 --setKey “null” --filteredAreUncalled --filteredrecordsmergetype KEEP_IF_ANY_UNFILTERED -o PON_celltype1.vcf.

Pair-wise comparison of sample versus panel of normals and identification of variants:

java -jar GenomeAnalysisTK.jar -T MuTect2 -R human_g1k_v37.fasta -I:tumor patient1_celltype1.bam --normal_panel PON_celltype2.vcf -nct 20 -o variants_patient1_celltype1.vcf.

#### VarScan2 (adapted settings)

Removal of duplicates:

java -Xmx10g -jar picard.jar MarkDuplicates I=patient1_celltype1.bam O=patient1_celltype1_dedup.bam M=patient1_celltype1_dedup_metrix.txt REMOVE_DUPLICATES=true.

Conversion to pileup format:

samtools mpileup -f human_g1k_v37.fasta -l regions.bed patient1_celltype1_dedup.bam > patient1_celltype1.pileup.

Pair-wise comparison of cell type within patient and identification of variants:

java -jar VarScan.v2.3.9.jar somatic patient1_celltype1.pileup patient1_celltype2.pileup patient1_2vs1_output --min-var-freq 0 --min-coverage 1 --min-reads2 1.

The overlap between callers was determined using the Linux command line tool join. The overlapping variants were annotated with Annovar software (Version June 2013) (46). The filtering based on the technical parameters consisted of retaining variants

- with a coverage of ≥300 in target and reference sample;
- with an AAF in target sample ≥0.5% and in reference sample <0.5%;
- that is observed max twice in the same cell type and max once in the other cell type;
- that are detected on both strands;
- passing all nine default MuTect2 filters/only failing *clustered events* or *homologous mapping event* filters.

Variants were further prioritized if they were

- SNVs located in exons or at splice sites;
- rare (≤1%) in public databases: 1000 genomes project 2015, exome sequencing project 6500 (ESP6500), complete genomics 69 (cg69), exome aggregation consortium (ExAC) and Known VARiants database (Kaviar);
- not located in segmental duplications;
- located in genes not frequently mutated in healthy population (GDI < 13.84%).

Criteria specifically related to the sequencing design were adapted for analysis of the RA data, as described in the results and in Supplementary Material, Table S2.

A Data Resource of called variants before filtering and prioritization is available through the Corresponding Author.

### Replication phase: replication of somatic variants

For replication, PCR amplicons for each of the putative somatic variants identified in the screening phase were prepared from DNA of the affected individual and pooled per cell type and sequenced using the MiSeq V3 technology (PE300) (LGC Genomics, Teddington, UK) or the NovaSeq technology (PE100) (CeGaT, Tübingen, Germany). Overlap between paired reads was determined with PandaSeq (version 2.11). Both reads with and without overlap were aligned with BWA software (version 0.7.12) (44) to the human Reference Genome Build (NCBI37/hg19) and sorted with SAMtools (47,48). Realignment of reads and base recalibration was performed by GATK (version 3.5) (45). Variant calling was done with VarScan2 (version 2.3.9) (21), with standard minimal sequencing depth, standard minimal reads supporting the variant, decreased minimal variant frequency of 0% and without quality score criteria.

For cloning-based replication, PCR amplicons were cloned with the TOPO TA Cloning Kit with PCR2.1-TOPO vector (Thermo Fisher Scientific) according to the standard protocol. Colonies containing the desired PCR fragment were isolated and amplified with a PCR using the appropriate primers, followed by Sanger sequencing (LGC Genomics).

For ddPCR, 50 ng DNA was cut with 2.5 U EcoRI restriction enzyme and 1× NEBuffer EcoRI (Bioké, Leiden, The Netherlands) in a reaction volume of 10 μl for 1 h at 37°C. Subsequently, ddPCR was performed according to the standard protocol (Bio-Rad, Hercules, California) using custom genotyping assays (LifeTechnologies, Carlsbad, California—sequences available upon request). An average of 13 000 droplets were generated in a 20 μl reaction, and data were analysed with the QuantaSoft software (version 1.7.4.0917, Bio-Rad). Plasmids carrying either the reference or alternate allele were constructed by inserting a PCR product into the PCR2.1 TOPO vector, as described above. Plasmids were isolated from these bacterial colonies using the Qiagen® Plasmid Mini Kit (Qiagen, Venlo, The Netherlands) according to the standard protocol. A standard curve with known amounts of plasmids carrying the reference and alternate allele was set up in order to determine the correlation between the expected values (based on the input) and observed values (based on ddPCR output).

### Germline variant calling

Matched germline variants were identified for comparison using the same in-house protocol. After performing alignment, base recalibration and local realignment as described above for the screening phase, SNVs were called with GATK Unified Genotype Caller (version 3.1.1). Variants were annotated with Annovar. Before comparing their characteristics to the confirmed somatic variants, the germline variants underwent filtering analogue to the filtering of somatic variants to correct for the biases the filtering introduces.

### T-cell receptor determination

Peripheral blood was collected in 4 ml blood tubes containing EDTA (BD Vacutainer). T-cell receptor subsets were determined as part of a standard hematological phenotyping (Laboratory medicine, UZ Leuven) with the addition of markers to distinguish CD4^+^ and CD8^+^ populations.

### Statistical analyses

Statistical analyses used for characterization and replication were performed using R (version 2.15.2) statistical software and using the statistical tests implemented in VarScan2 (21). The significance thresholds were corrected for multiple testing.

Tests for independence (variants in B versus T cells, synonymous versus non-synonymous variants) and comparisons of categorical variables between two groups (replicated versus non-replicated variants, somatic versus germline variants) were performed using the Fisher’s exact test. Differences in continuous variables between two groups were tested by the Welch two-sample *t*-test (replicated versus non-replicated variants) or the Kruskal Wallis test (somatic versus germline variants) after testing for normal distribution of the data with the Shapiro–Wilk test. Difference from zero for a single continuous variable (clonal expansion rate) was tested by the Wilcoxon signed rank test. Spearman’s rank correlation test (AAF screening versus replication phase) or Pearson product–moment correlation (variant count versus patient’s age or disease duration) was used to test correlations between continuous variables after testing for normal distribution of the data with the Shapiro–Wilk test.

## Acknowledgements

The authors thank the patients for participating in this study; C. Thys and K. Clysters for assistance in sample collection and management; Dr M. Moisse for bioinformatics assistance, critical revision and discussion of the manuscript and Prof X. Bossuyt for hematological phenotyping.

*Conflict of Interest statement.* None declared.

## Funding

Research Council KU Leuven (C24/16/045, CREA14/023 to A.G. and B.D.); Research Foundation Flanders (G073415, G0A7219N to A.G. and Clinical Investigatorship to B.D.); Belgian Charcot Foundation (to A.G., PhD Fellowship to L.V.H.); MS Liga Vlaanderen (to A.G.); Queen Elisabeth Medical Foundation (to A.G.); VSC (Flemish Supercomputer Center) funded by the Research Foundation—Flanders (FWO) and the Flemish Government – department EWI; European Research Council (M-IMM project) (to S.M.); Academy of Finland (to S.M.); Finnish special governmental subsidy for health sciences, research and training (to S.M.); Sigrid Juselius Foundation (S.M.).

## Supplementary Material

VanHorebeek_SupplData_Final_ddy425
