# Supplementary material for: A robust pipeline with high replication rate for detection of somatic variants in the adaptive immune system as a source of common genetic variation in autoimmune disease
Source: Hum Mol Genet. 2018 Dec 12;28(8):1369–80. doi: 10.1093/hmg/ddy425 (PMC6452186; doi:10.1093/hmg/ddy425)
Supplement: VanHorebeek_SupplData_Final_ddy425 [file vanhorebeek_suppldata_final_ddy425.pdf]

Supplementary data

Development of a robust pipeline for detection of somatic variants in the adaptive immune system identifies a novel class of genetic variation in multiple sclerosis

Lies Van Horebeek[1,7], Kelly Hilven[1,7], Klara Mallants[1], Annemarie Van Nieuwenhuijze[2,3], Paula Savola[4,5], Tiina Kelkka[4,5], Satu Mustjoki[4,5], Susan M. Schlenner[2,3], Adrian Liston[2,3], Bénédicte Dubois[1,6], An Goris[1,*]

Supplementary table 1. Details of immune cell selection and sequencing per patient
Supplementary table 2. Overview filtering criteria
Supplementary table 3. Details of exclusion reasons of false positives in validation dataset
Supplementary table 4. Systematic analysis of pipelines in validation dataset
Supplementary table 5. Genes with high GDI contribute disproportionally to somatic variants in MS
Supplementary table 6. Overview of candidate somatic variants in MS
Supplementary figure 1. Example of gating strategy and purity of immune cell selection using flow cytometry
Supplementary figure 2. Associations between parameters and replication status of the somatic variants
Supplementary figure 3. Distribution of the nucleotide changes for replicated variants
Supplementary figure 4. T cell receptor pattern MS-3
Supplementary figure 5. Immunophenotyping results of T cell subsets for MS-3
Supplementary figure 6. ddPCR standard curve
Supplementary figure 7. ddPCR method

Supplementary table 1. Details of immune cell selection and sequencing per patient

	B			T		
	**Cell Count**	**DNA (ng)**	**Sequencing depth**	**Cell Count**	**DNA (ng)**	**Sequencing depth**
**MS-1**	128,296	356	296	136,755	448	336
**MS-2**	185,699	609	405	54,844	420	205
**MS-3**	255,895	464	320	580,728	1,659	388
**MS-4**	171,917	294	252	138,608	351	363
**MS-5**	571,051	2,920	349	219,419	1,199	309
**MS-6**	19,154	480	163	48,245	255	232
**MS-7**	194,741	584	411	93,316	240	363
**MS-8**	170,869	486	282	149,985	788	278
**MS-9**	191,722	626	348	193,196	694	368
**MS-10**	259,078	721	367	351,194	1,370	428

Table representing the amount of cells that were sorted by FACS, the amount of DNA that was extracted from these cells and used as input for the screening phase and the average sequencing depth obtained during the screening phase for each patient for B cells (columns 2-4) and T cells (columns 5-7) respectively.

Supplementary table 2. Overview filtering criteria

**Filtering criteria MS dataset**	**Filtering criteria RA dataset**
Technical parameters	
Detected by MuTect2 and VarScan2	Detected by MuTect2 and VarScan2
Coverage ≥ 300	Coverage ≥ 150
AAF target ≥ 0.5%, AAF reference < 0.5%	AAF target ≥ 1%, AAF reference < 0.5%
Max 2x PON target, max 1x PON reference	Max 6x PON target, max 2x PON reference
Variant detected on both strands	Variant detected on both strands
Filter MuTect2[a]	Filter MuTect2[a]
Prioritization parameters	
SNVs in exons/splice sites	SNVs in exons/splice sites
Rare	Rare
Not in segmental duplications	Not in segmental duplications
Gene not frequently mutated in healthy population: GDI < 13.84	Gene not frequently mutated in healthy population: GDI < 13.84

Overview of filtering criteria for MS (left) and RA (right) dataset. AAF: alternate allele fraction, PON: panel of normal, SNV: single nucleotide variant, GDI: gene damage index.

[a] Originally, variants were required to pass all nine default MuTect2 filters. As application on an independent dataset showed that this was too stringent, variants are allowed to fail the filters *clustered events* and *homologous mapping event* in our final filtering.

Supplementary table 3. Systematic analysis of pipelines in validation dataset

Pipeline	True Positive or Replication Rate	
	True Positive/ (True Positive + False Positive)	%
**VarScan2 only** [a]	7/49	14.3
**Varscan2 + technical filtering**	7/18	38.9
**MuTect2 + VarScan2**	7/12	58.3
**MuTect2 + VarScan2 + technical filtering**	7/8	87.5

[a] These are the results of Savola *et al*. *Nature Communications* 2017 based on the Varscan2 tool only. As this tool is used to generate the list of known positive and negative variants in the independent validation dataset, it is not possible to omit this tool for a comparison of Mutect2 only.

Supplementary table 4. Details of exclusion reasons of false positives in validation dataset

Filtering criterion	Number of variants failing criterion (N=41)	Number of variants uniquely failing criterion (N=10)
**Passing MuTect2 filters**	36	8
**Plausible occurrence across samples**	26	0
**Not located in segmental duplication**	20	0
**Plausible alternate allele fraction in reference cell type**	15	0
**Sufficient coverage**	6	2
**Detection by MuTect2**	1	0
**No strand bias**	1	0

List of filtering criteria for which false positives variants from the RA validation dataset failed. Column 1 indicates which filtering criteria the false positives failed. Column 2 and 3 indicate the total number of false positives failing the criterion and the number of variants for which the filtering criterion is the only criterion causing exclusion.

Supplementary table 5. Genes with high GDI contribute disproportionally to somatic variants in MS

	Total	High GDI	Percentage
**Confirmed somatic variants**	43	7	16.28%
**Candidate genes**	5,899	309	5.24%

Supplementary table 6. Overview of candidate somatic variants in MS

	Chr	Position	Gene	RefSeq ID	Base change	AA change	Cell	Screening				Replication				
								Ref in target	Alt in target	Ref in reference	Alt in reference	Ref in target	Alt in target	Ref in reference	Alt in reference	p
**MS-1**																
*	**2**	**24952447**	***NCOA1***	**NM_003743**	**C2964T**	**P988P**	**T**	**442**	**9**	**372**	**0**	**151937**	**878**	**150517**	**38**	**9.60E-207**
	2	135119956	*MGAT5*	NM_002410	G1357T	G453C	B	733	6	726	1	67037	174	29142	77	0.58
*	2	155555854	*KCNJ3*	NM_001260508	G567T	K189N	T	824	9	866	1	139335	253	85616	147	0.31
	**3**	**48508943**	***TREX1***	**NM_016381**	**C1054G**	**L352V**	**T**	**525**	**15**	**591**	**0**	**41755**	**1083**	**28643**	**81**	**1.00E-149**
	5	121758799	*SNCAIP*	NM_001308105	G367T	G123X	T	670	6	605	0	30005	-[a]	35232	-[a]	-[a]
*	**11**	**8734271**	***ST5***	**NM_139157**	**C739T**	**R247C**	**T**	**926**	**7**	**866**	**3**	**125917**	**1070**	**124673**	**28**	**1.41E-272**
*	15	52556415	*MYO5C*	NM_018728	C1019A	A340E	T	1268	7	1093	1	243387	451	223889	466	0.96
	**19**	**49703983**	***TRPM4***	**NM_001321285**	**G1832A**	**R611H**	**T**	**675**	**11**	**736**	**1**	**42101**	**477**	**113414**	**304**	**2.67E-87**
MS-2																
	1	167823649	*ADCY10*	NM_001167749	C1791A	L597L	B	613	5	344	0	235360	508	335682	668	0.09
	2	212543831	*ERBB4*	NM_001042599	G1568T	C523F	T	574	6	1492	0	155686	135	354475	386	0.99
	3	57132269	*IL17RD*	NM_017563	G1462A	V488I	B	1152	7	548	0	201415	-[a]	246199	-[a]	-[a]
	3	184105212	*CHRD*	NM_001304472	C2398T	H800Y	T	379	7	521	2	66681	-[a]	22588	-[a]	-[a]
	11	78369111	*TENM4*	NM_001098816	C8302A	R2768R	B	915	5	480	1	137240	304	178432	354	0.09
MS-3																
*	**1**	**93202076**	***EVI5***	**NM_001308248**	**A160G**	**T54A**	**T**	**406**	**4**	**337**	**1**	**54833**	**632**	**54274**	**77**	**8.70E-108**
*	1	221057559	*HLX*	NM_021958	G980A	R327Q	T	755	8	489	0	168530	-[a]	148291	-[a]	-[a]
*	**2**	**33590430**	***LTBP1***	**NM_001166264**	**A3467T**	**D1156V**	**T**	**2142**	**15**	**1482**	**0**	**111314**	**897**	**100824**	**304**	**3.71E-56**
*	2	208992963	*CRYGC*	NM_020989	G489A	K163K	T	1243	8	873	0	275567	-[a]	302044	[a] -	-[a]
*	**3**	**46244852**	***CCR1***	**NM_001295**	**G953C**	**R318T**	**B**	**485**	**6**	**670**	**2**	**29762**	**425**	**109904**	**186**	**5.80E-143**
*	5	33989483	*AMACR*	NM_001167595	C864T	D288D	T	1601	8	1174	1	198703	-[a]	180123	-[a]	-[a]
*	**6**	**74073541**	***KHDC3L***	**NM_001017361**	**G612A**	**Q204Q**	**T**	**1023**	**61**	**999**	**0**	**60900**	**2386**	**75707**	**11**	**0**
*	**11**	**63403722**	***ATL3***	**NM_001290048**	**A881G**	**N294S**	**T**	**467**	**12**	**417**	**0**	**264400**	**7390**	**252116**	**739**	**0**
*	**11**	**128680557**	***FLI1***	**NM_001271012**	**A454G**	**K152E**	**T**	**683**	**8**	**500**	**1**	**117694**	**1727**	**120325**	**226**	**2.20E-291**
*	**12**	**123812503**	***SBNO1***	**NM_001167856**	**C1368G**	**G456G**	**T**	**2521**	**20**	**1610**	**7**	**125845**	**7440**	**136039**	**1362**	**0**
*	**16**	**67116210**	***CBFB***	**NM_001755**	**A494T**	**E165V**	**B**	**371**	**7**	**535**	**0**	**41039**	**1176**	**49934**	**294**	**2.10E-163**

*****	**Y**	**16734258**	***NLGN4Y***	**NM_001164238**	**C259T**	**R87W**	**B**	**831**	**9**	**1119**	**2**	**23766**	**184**	**21127**	**90**	**1.33E-6**
MS-4																
*****	**16**	**88504410**	***ZNF469***	**NM_001127464**	**T10448C**	**L3483P**	**T**	**396**	**5**	**547**	**1**	**184576**	**8261**	**79840**	**3245**	**2.38E-6**
MS-5																
*	1	228362933	*IBA57*	NM_001010867	G790T	E264X	T	1324	11	1557	1	30347	-[a]	20274	-[a]	-[a]
*	12	21919301	*KCNJ8*	NM_004982	C631A	R211R	B	738	5	679	1	154932	306	221837	436	0.49
*	14	61115604	*SIX1*	NM_005982	C304A	P102T	T	338	4	359	0	25010	154	12727	103	0.99
MS-7																
*	3	133670090	*SLCO2A1*	NM_005630	T823C	F275L	T	325	5	422	0	55358	-[a]	169225	-[a]	-[a]
*	8	27462493	*CLU*	NM_001831	C777A	D259E	T	1057	7	1447	0	82005	92[b]	36926	61[b]	8.34E-47[b]
*	11	132527170	*OPCML*	NM_001319103	G212A	R71H	T	680	5	739	1	34086	-[a]	85953	-[a]	-[a]
*	17	60045509	*MED13*	NM_005121	C4078A	L1360M	B	1105	6	840	1	107756	318	67922	184	0.19
*	17	74625545	*ST6GALNAC1*	NM_018414	C380A	P127Q	B	330	7	355	0	20202	443	14495	248	0.001
MS-8																
	1	155448319	*ASH1L*	NM_018489	C4342A	R1448R	T	581	6	486	0	301127	622	221000	482	0.82
	2	44428873	*PPM1B*	NM_001033557	C535T	R179X	B	311	6	397	0	345582	-[a]	232420	-[a]	-[a]
	**2**	**135888230**	***RAB3GAP1***	**NM_001172435**	**G1175A**	**R392Q**	**T**	**1129**	**8**	**1497**	**1**	**69506**	**355**	**68000**	**204**	**4.90E-10**
	**6**	**31631776**	***GPANK1***	**NM_033177**	**C480G**	**S160R**	**T**	**548**	**123**	**1007**	**1**	**141509**	**22808**	**120600**	**431**	**0**
	**6**	**149700524**	***TAB2***	**NM_001292034**	**T1473C**	**L491L**	**T**	**349**	**8**	**320**	**0**	**304190**	**7954**	**245232**	**266**	**0**
	**7**	**47409021**	***TNS3***	**NM_022748**	**C1222T**	**R408C**	**T**	**913**	**9**	**1270**	**0**	**172207**	**1113**	**121687**	**42**	**1.99E-197**
	**8**	**135521904**	***ZFAT***	**NM_001174157**	**G3264T**	**S1088S**	**B**	**626**	**4**	**394**	**0**	**260967**	**1807**	**367645**	**1226**	**1.19E-88**
	10	70645216	*STOX1*	NM_001130161	C1664G	P555R	T	698	10	576	0	74942	-[a]	71575	-[a]	-[a]
	**16**	**67517193**	***AGRP***	**NM_001138**	**G109A**	**A37T**	**B**	**592**	**13**	**444**	**2**	**3401**	**65**	**34791**	**130**	**2.52E-21**
	**21**	**15538710**	***LIPI***	**NM_001302998**	**C706G**	**P236A**	**T**	**451**	**83**	**478**	**1**	**248632**	**49415**	**245384**	**187**	**0**
	X	153129390	*L1CAM*	NM_001143963	C3390A	L1130L	B	1039	8	766	0	77660	135	100548	176	0.55
MS-9																
	1	235922570	*LYST*	NM_000081	C6583A	L2195M	T	337	4	333	0	47521	166	49989	248	2.58E-3
	**1**	**240071069**	***CHRM3***	**NM_000740**	**C318T**	**F106F**	**T**	**409**	**33**	**377**	**0**	**271463**	**10340**	**191983**	**30**	**0**
	**2**	**97427889**	***CNNM4***	**NM_020184**	**A1153T**	**M385L**	**B**	**1155**	**14**	**1255**	**2**	**85016**	**1452**	**101402**	**263**	**1.20E-245**
	**5**	**133481460**	***TCF7***	**NM_001134851**	**T759C**	**D253D**	**T**	**529**	**6**	**548**	**0**	**284909**	**2321**	**177224**	**24**	**0**
	11	67939106	*KMT5B*	NM_001300909	C655A	L219I	T	456	7	533	0	29387	174	44964	305	0.93

**16**	**61891025**	***CDH8***	**NM_001796**	**C665T**	**T222I**	**T**	**1708**	**67**	**1836**	**2**	**26870**	**935**	**49302**	**163**	**4.30E-252**
X	115304399	*AGTR2*	NM_000686	G866T	S289I	T	469	5	453	0	141097	633	136677	874	5.48E-12
**X**	**29972647**	***IL1RAPL1***	**NM_014271**	**G1210T**	**D404Y**	**T**	**380**	**5**	**348**	**0**	**288229**	**2295**	**236291**	**339**	**4.60E-276**
MS-10															
2	60773289	*BCL11A*	NM_018014	C202A	R68R	T	397	5	364	0	273179	398	209864	342	0.94
**2**	**141533745**	***LRP1B***	**NM_018557**	**G5422A**	**G1808R**	**T**	**393**	**11**	**360**	**0**	**168427**	**3390**	**126343**	**26**	**0**
**6**	**26508797**	***BTN1A1***	**NM_001732**	**C976A**	**R326R**	**T**	**977**	**15**	**808**	**0**	**238860**	**3833**	**175019**	**466**	**0**
**7**	**103048325**	***SLC26A5***	**NM_001167962**	**G861A**	**P287P**	**T**	**498**	**6**	**403**	**0**	**218450**	**3354**	**156459**	**64**	**0**
7	106877083	*COG5*	NM_181733	A1933C	K645Q	T	1004	68	903	0	295701	-[a]	220891	-[a]	-[a]
**7**	**122635510**	***TAS2R16***	**NM_016945**	**A179C**	**Q60P**	**T**	**2075**	**81**	**1717**	**1**	**71208**	**2881**	**85566**	**331**	**0**
**9**	**73477936**	***TRPM3***	**NM_001007471**	**G350A**	**R117Q**	**T**	**1897**	**10**	**1610**	**0**	**236652**	**1523**	**214682**	**435**	**8.80E-119**
**11**	**118373702**	***KMT2A***	**NM_001197104**	**A7095C**	**K2365N**	**T**	**1290**	**21**	**1111**	**0**	**58384**	**1021**	**42777**	**375**	**9.15E-33**
**12**	**2622058**	***CACNA1C***	**NM_000719**	**A1298T**	**D433V**	**T**	**358**	**11**	**321**	**1**	**80681**	**3217**	**101821**	**234**	**0**
**17**	**65026679**	***CACNG4***	**NM_014405**	**C543T**	**Y181Y**	**T**	**513**	**46**	**518**	**0**	**228116**	**20471**	**174137**	**59**	**0**
**19**	**37618680**	***ZNF420***	**NM_001329516**	**A787C**	**N263H**	**T**	**1119**	**7**	**986**	**1**	**146042**	**900**	**121797**	**353**	**3.24E-36**
20	40944370	*PTPRT*	NM_007050	C2132A	A711D	T	468	5	423	2	55963	195	53186	256	3.87E-3
**X**	**17746076**	***NHS***	**NM_001291868**	**G3256T**	**D1086Y**	**B**	**968**	**9**	**1251**	**1**	**51018**	**245**	**71443**	**233**	**1.52E-05**

List of putative somatic variants resulting from our final pipeline grouped per patient. '*' indicates that a second sample obtained at a later time point was used for the replication phase. Replicated somatic variants are indicated in bold. Column 4 represents the RefSeq ID of the transcript for which the base change and amino acid change are given in columns 5 and 6, respectively. Column 7 represents the cell type in which the somatic variant was identified. Columns 8-11 represent the number of reads with the reference allele in B cells, alternate allele in B cells, reference allele in T cells and alternate allele in T cells for the screening phase. Columns 12-15 represent the same for the replication phase. Column 16 represents the VarScan2 p-value obtained during the replication pahse in support of a somatic variant.

[a] The alternative allele of the screening phase was not recognized by VarScan2 as the alternative allele during the replication phase.

[b] The alternate allele fraction is lower in the reference tissue than in the target tissue, thus the variant is not replicated.

Chr: chromosome, Ref: reference allele, Alt: alternate allele, AA change: amino acid change, p: p-value.

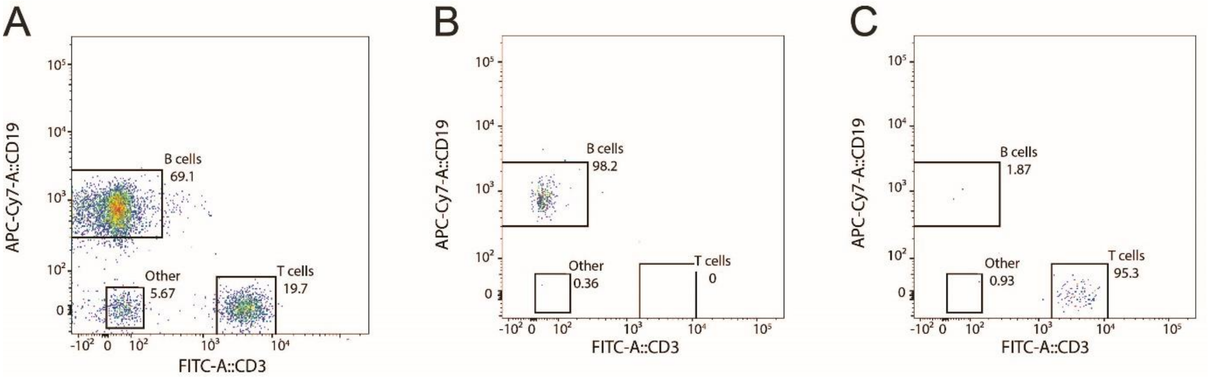

Supplementary figure 1. Example of gating strategy and purity of immune cell selection using flow cytometry

(A) Before sorting, (B) B cells and (C) T cells after sorting: purity >95% is reached. The axes indicate the dyes used to distinguish the different cell types.

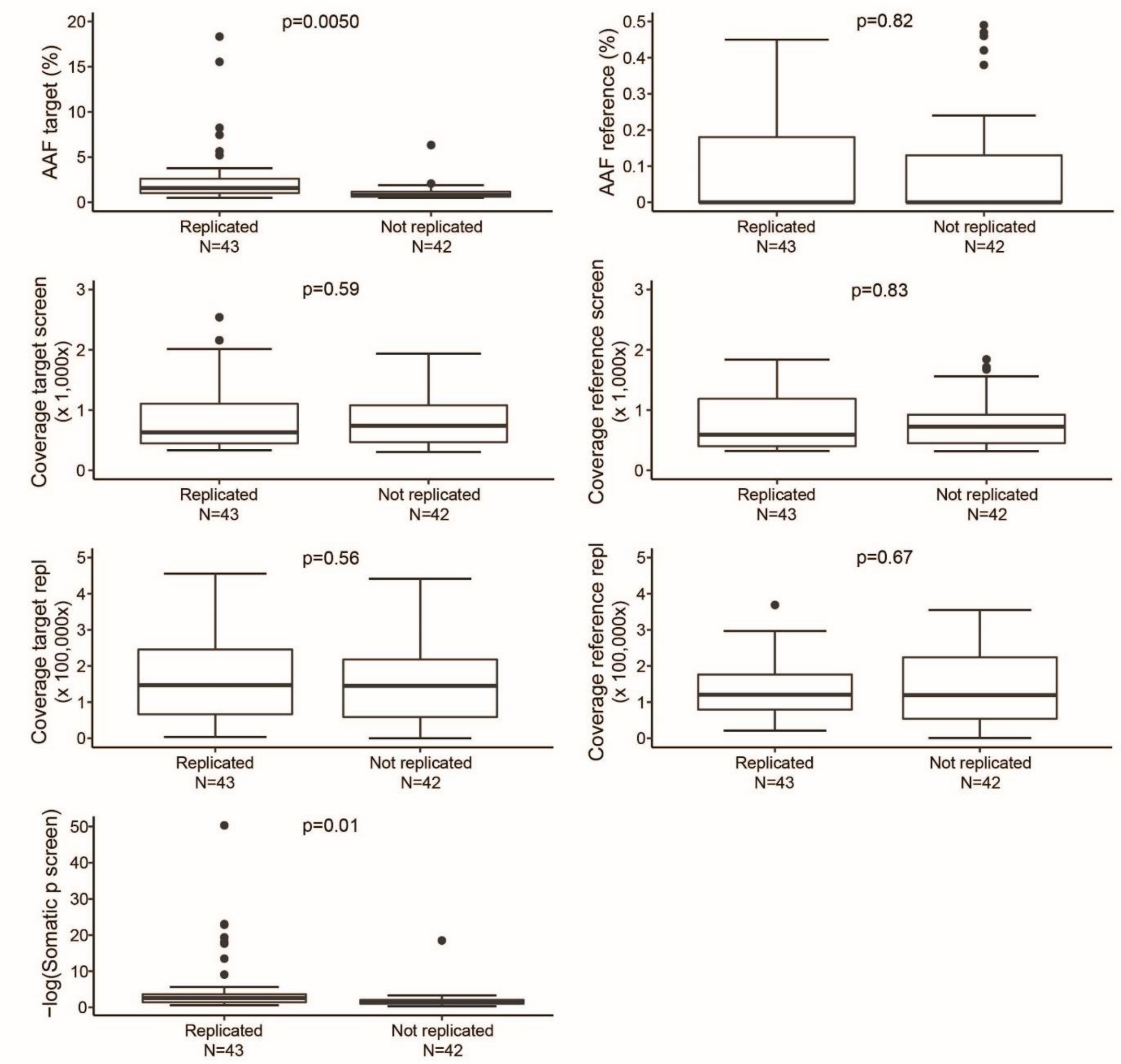

Supplementary figure 2. Associations between parameters and replication status of the somatic variants (A,B) Alternate allele fraction of the variant in the (A) target and (B) reference sample obtained from the screening phase. (C-F) Sequencing depth at location of variant in (C,E) target sample and (D,F) reference sample during (C,D) screening phase and (E,F) replication phase. (G) Somatic p-value calculated by VarScan2 based on the screening phase.

AAF: alternate allele fraction, screen: screening phase, repl: replication phase.

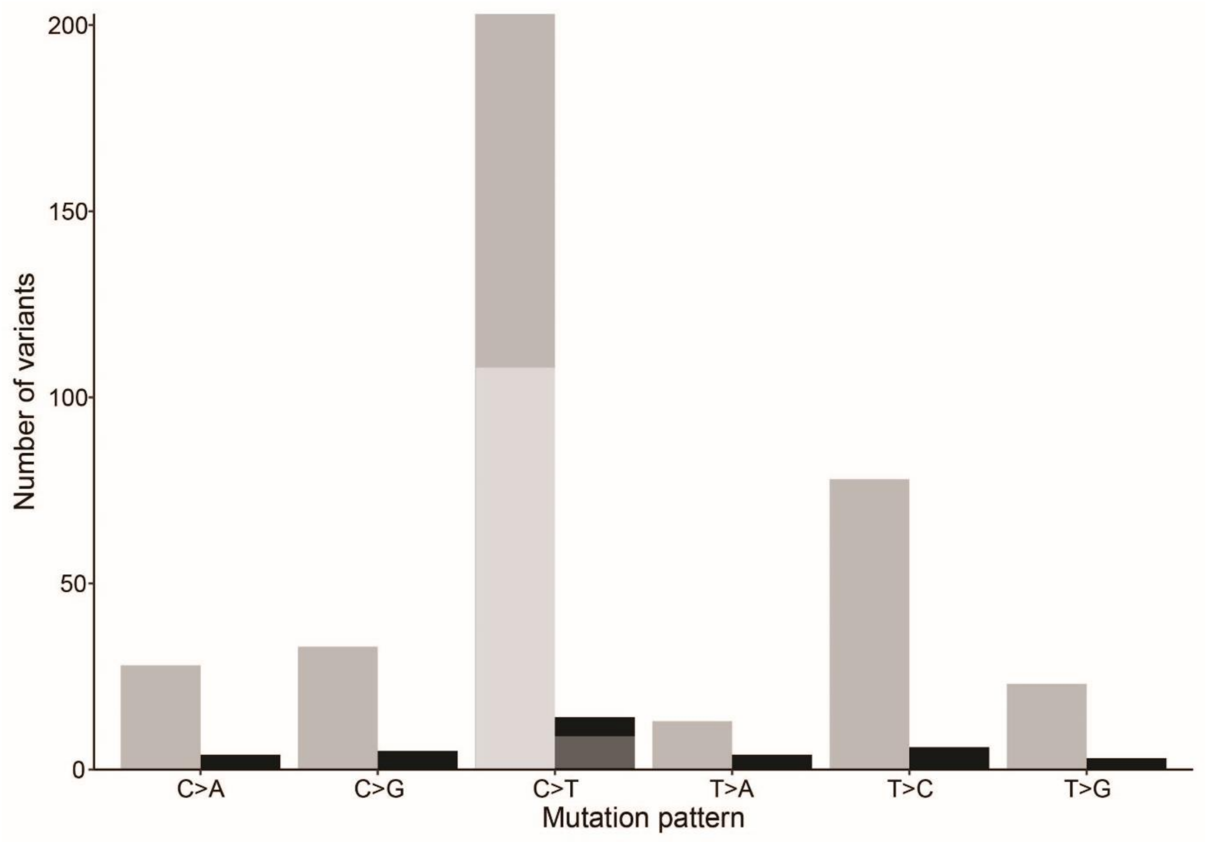

Supplementary figure 3. Distribution of the nucleotide changes for replicated variants

Chi-square goodness of fit: p=0.076. Fisher-Exact for CpG>TpG: p=0.58. The number of germline variants are indicated in grey, the number of somatic variants in black. Lighter shades for C>T indicate the number of variants located at CpG positions.

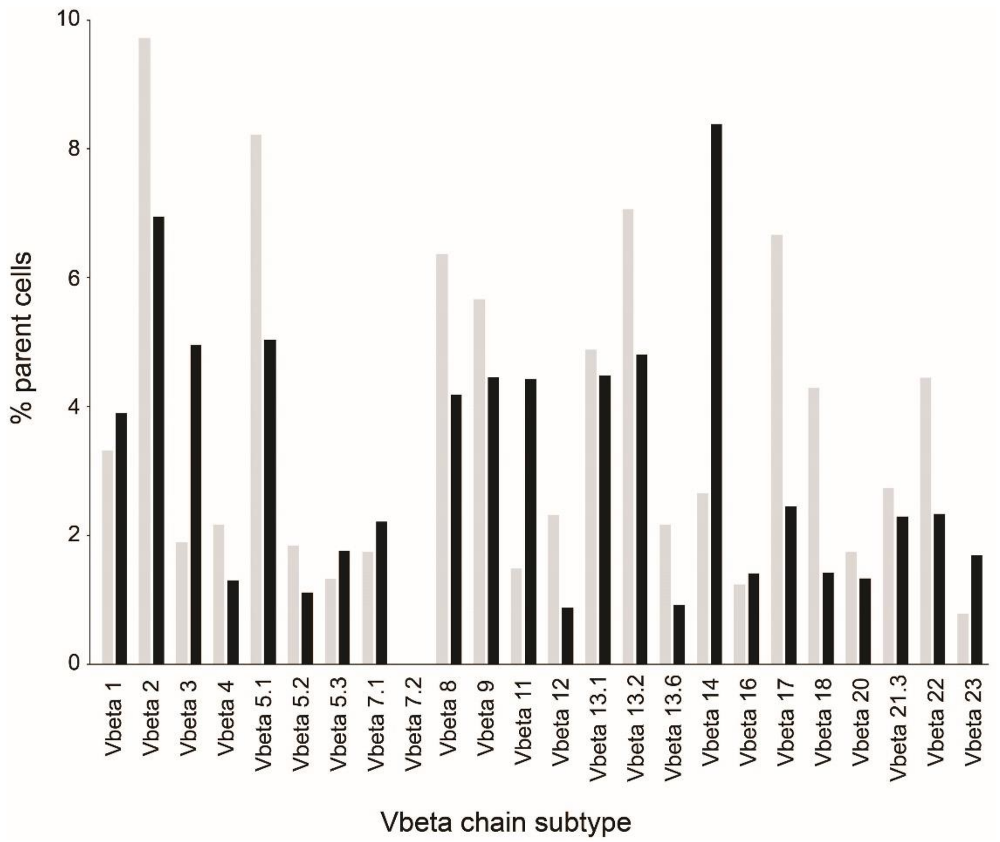

Supplementary figure 4. T cell receptor pattern MS-3

T cell receptor pattern of $CD4^+$ T cells indicated in grey and of $CD8^+$ T cells indicated in black for MS-3 as determined by hematological phenotyping. Percentages are given compared to the parent cells, being $CD4^+$ T cells or $CD8^+$ T cells.

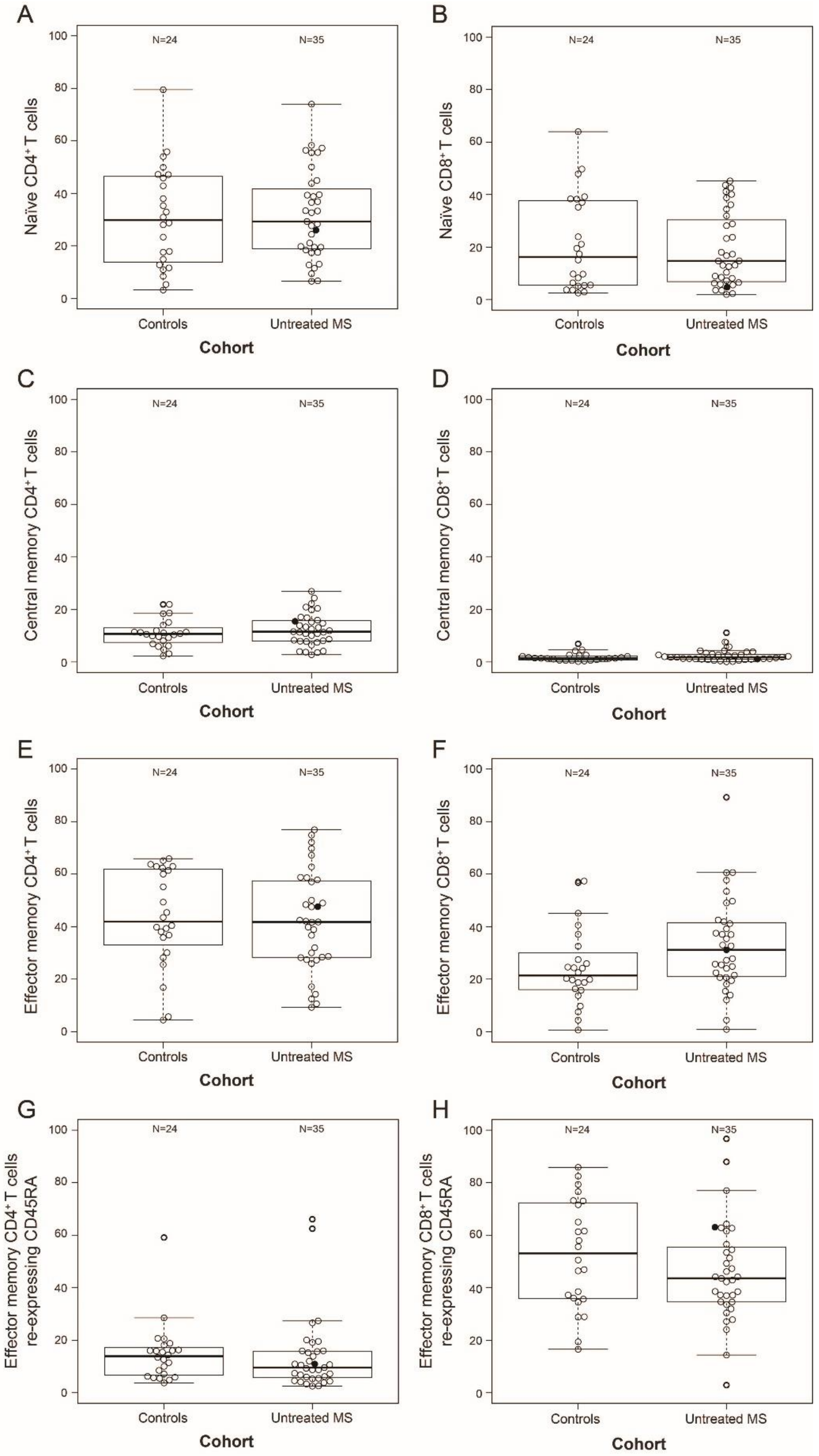

Supplementary figure 5. Immunophenotyping results of T cell subsets for MS-3 Boxplots representing T cell subsets in controls and untreated MS patients. Naïve (A), central memory (C), effector memory (E) and effector memory T cells re-expressing CD45RA (G) $CD4^+$ T cells (as percentage of all $CD4^+$ T cells) and naïve (B), central memory (D), effector memory (F) and effector memory T cells re-expressing CD45RA (H) $CD8^+$ T cells (as percentage of all $CD8^+$ T cells) are presented. MS-3, followed up in detail is indicated as a black dot in the untreated MS cohort.

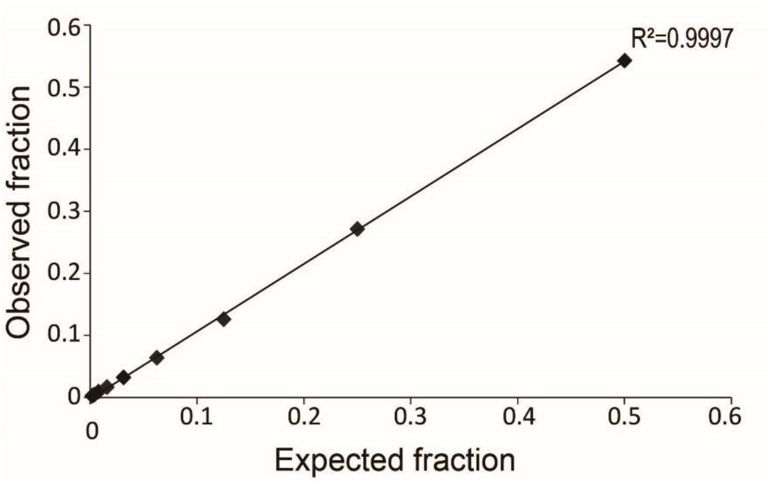

Supplementary figure 6. ddPCR standard curve

Graph indicating the correlation between the theoretically expected fraction of the alternate allele and the actual fraction measured by ddPCR. A very high correlation (r^2=0.9997) is observed between the observed and expected fraction of the alternate C-allele. Each point is the average of two duplicate measurements, with r^2=0.98 between duplicates.

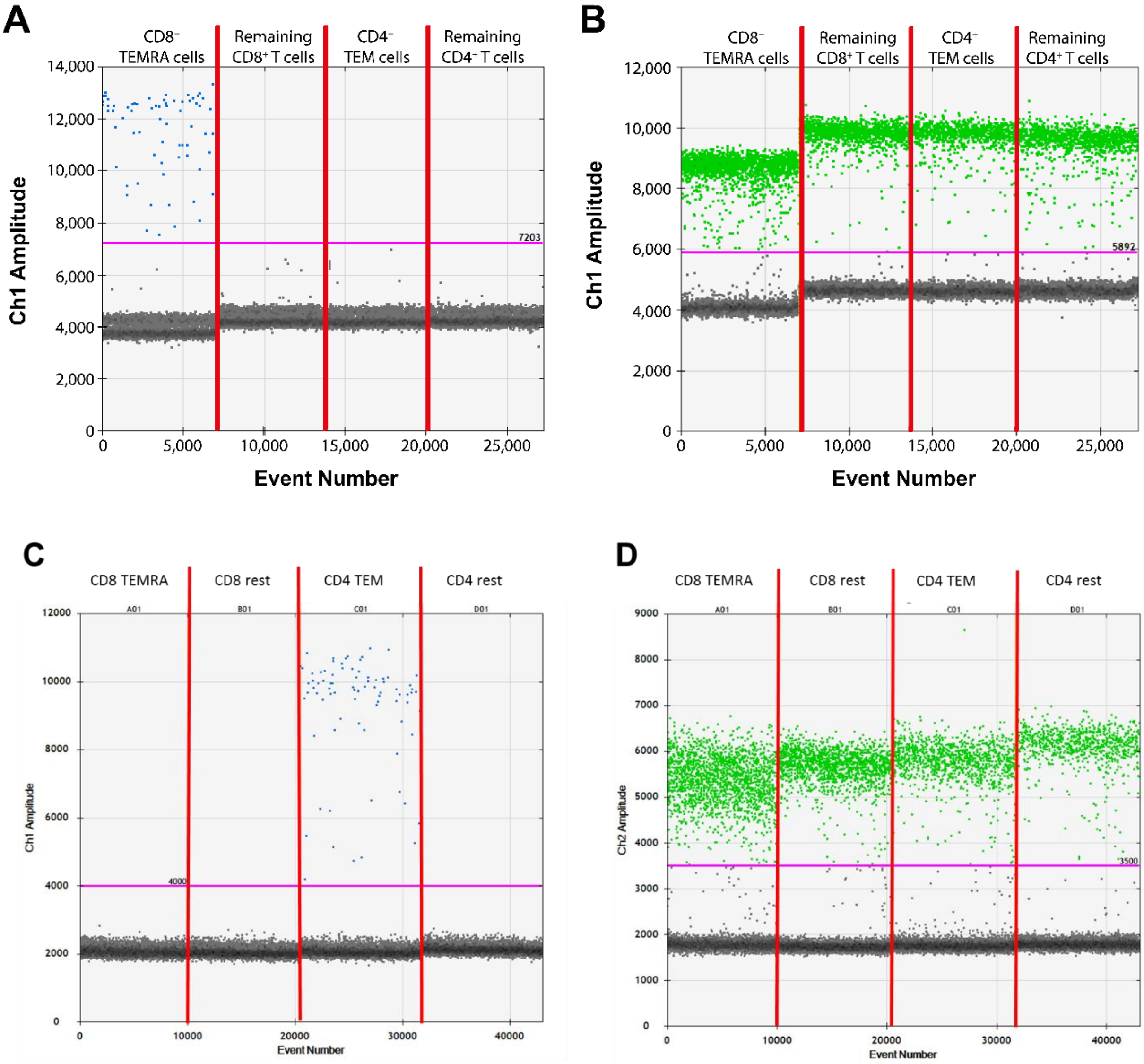

Supplementary figure 7. ddPCR method

Figure representing the number of droplets in a total volume of 20 µl positive for (A,C) the alternate allele (shown as blue dots) and (B,D) the reference allele (shown as green dots) in DNA obtained from T cell subsets (TEMRA and all other $CD8^+$ T cells, TEM and all other $CD4^+$ T cells) of MS patient MS-3 for variants in *EVI5* (A,B) and *LTBP1* (C,D). Negative droplets are indicated in grey, and the threshold for positive droplets is indicated in pink. The alternate alleles are solely observed in $CD8^+$ TEMRA and $CD4^+$ TEM cells, respectively.
